# CLCA4 inhibits bladder cancer cell proliferation, migration, and invasion by suppressing the PI3K/AKT pathway

**DOI:** 10.18632/oncotarget.21724

**Published:** 2017-10-09

**Authors:** Teng Hou, Lijie Zhou, Longwang Wang, Gallina Kazobinka, Xiaoping Zhang, Zhaohui Chen

**Affiliations:** ^1^ Department of Urology, Union Hospital, Tongji Medical College, Huazhong University of Science and Technology, Wuhan, Hubei 430022, China; ^2^ Department of Urology, The Second Affiliated Hospital of Nanchang University, Nanchang, Jiangxi 330008, China

**Keywords:** CLCA4, bladder cancer, PI3K/Akt, proliferation, metastasis

## Abstract

Calcium activated chloride channel A4 (CLCA4), a tumor suppressor, was shown to contribute to the progression of several human cancers, while its role in bladder carcinoma remains unclear. In this study, we showed CLCA4 expression was down-regulated in bladder carcinoma tissues and cells compared to adjacent non-tumor tissues and normal urothelial cells. Low CLCA4 expression was correlated with larger tumor size, advanced tumor stage, and poor prognosis in bladder carcinoma patients. Overexpression of CLCA4 profoundly attenuated the proliferation, growth, migratory and invasive capabilities of bladder cancer cells, whereas CLCA4 knockdown had the opposite effect. Mechanistically, CLCA4 is involved in PI3K/AKT signaling and its downstream molecules can promote bladder cancer cell proliferation. Additionally, CLCA4 could mediate the migration and invasion of bladder cancer cells by regulating epithelial-mesenchymal transition and PI3K/Akt activation. This study suggests that CLCA4 may represent a promising prognostic biomarker for bladder cancer and provides a possible mechanism for bladder cancer growth and invasion.

## INTRODUCTION

Bladder cancer represents the most common cancer of the urinary tract. Overall, the annual incidence rate is 80.5/100,000 in China, with an estimated mortality rate of 32.9/100,000 [1]. Although the clinical treatment outcomes for bladder cancer have improved in recent decades, the survival time of patients with invasive stage tumors or metastatic diseases remains very short. In particular, the median survival time of these patients is about 14 months, while the long-term disease-free survival rate is only 15% [2]. Although tumor progression and migration are the most ruinous aspects of bladder cancer and directly impact the clinical survival of patients, the underlying mechanisms of these processes remain elusive. Therefore, it is necessary to investigate the underlying mechanisms of bladder cancer development and identify effective therapeutic strategies to improve patient survival.

Calcium-activated chloride channel (CLCA) regulators are a family of proteins that are characterized by a symmetrical multiple cysteine motif in the amino-terminal tail [3]. The human CLCA locus is located on chromosome 1p31-1p22 [4]. As outwardly rectified chloride channel modulators, CLCA proteins are activated by calcium to play a role in chloride conductance in epithelial cells [5]. In mammals the CLCA protein family members are distinguished by the von Willebrand factor type A domain, the structure of metalloprotease, and the capability to self-cleave [6]. Studies have shown that CLCA members were involved in a diverse range of biological processes including cell differentiation, adhesion, apoptosis, and the development of inflammatory processes in the airway [7]. In addition, some reports have revealed involvement of CLCA1, CLCA2, and CLCA4 in tumor progression. CLCA1 may contribute to the increased spontaneous differentiation and reduced cell proliferation of colorectal cancer [8], and is thus associated with favorable prognosis of colorectal cancer patients [9]. As a p53-inducible senescence regulator, CLCA2 negatively mediates tumor cell invasion [10], while loss of CLCA2 facilitates epithelial-to-mesenchymal transition and implicates higher chance of cancer metastasis [11].

CLCA4 is a member of the CLCA family, and the primary structure of CLCA4 is similar to CLCA1 and 2 [12]. RT-PCR analysis revealed that high levels of CLCA4 mRNA is expressed in human brain, testis, small intestine, colon and lung tissues [7]. Recently, CLCA4 was found to be aberrantly expressed in breast cancer, and its ectopic expression inhibited tumour cell growth as well as the epithelial to mesenchymal transition [13]. However, the impact of CLCA in bladder cancer development still needs to be verified. In this study, we examined the role and mechanism of CLCA4 in human bladder cancer. We demonstrated that CLCA4, whose low expression associates with tumor aggressiveness and unfavorable clinical survival, promoted bladder cancer cell proliferation and metastasis via the PI3K/AKT pathway. Together with other published data, our results showed that CLCA4 might be exploited as a target for potential clinic treatments of patients with bladder cancer.

## RESULTS

### CLCA4 expression is down-regulated in bladder cancer

**Figure 1:**
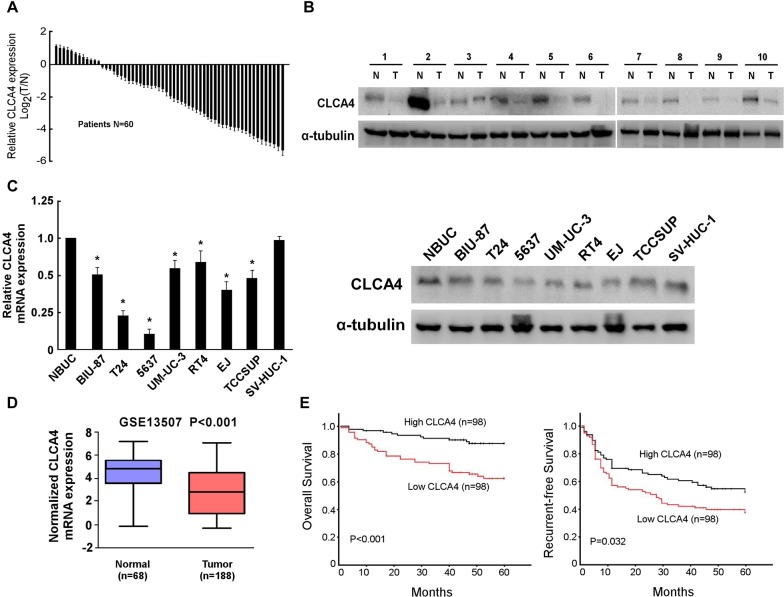
CLCA4 expression is decreased in bladder cancer **(A)** CLCA4 mRNA expression in 60 pairs of bladder cancer and adjacent non-tumor tissues. **(B)** The protein expression of CLCA4 in 10 pairs of randomly selected tumor (T) and adjacent non-tumor tissues (N). **(C)** CLCA4 mRNA and protein in normal bladder urothelial cells (NBUCs), bladder cancer cell lines (BIU-87, T24, 5637, UM-UC-3, RT4, EJ, and TCCSUP), and human uroepithelial cells (SV-HUC-1). **(D)** The expression levels of CLCA4 mRNA in tumor and normal tissues of bladder cancer patients from GSE13507 database. **(E)** Kaplan–Meier analysis of overall survival stratified by low CLCA4 expression (n = 98) and high CLCA4 expression (n = 98). CLCA4 up-regulation was significantly correlated with more favorable overall (P<0.001) and recurrent-free survival (P=0.032).

To investigate whether CLCA4 expression is correlated with bladder cancer development and progression, we first analyzed CLCA4 expression in bladder cancer cell lines and tissues. Decreased CLCA4 mRNA expression was detected in 80.0% (48/60) of the patients (Figure 1A). Additionally, western blot analysis showed that CLCA4 expression was markedly lower in bladder cancer than that in adjacent non-tumor tissues (Figure 1B). In addition, both western blot (WB) and real-time PCR analyses showed that CLCA4 was profoundly down-regulated in all 7 bladder cancer cell lines compared with NBUCs and normal urothelial cells (Figure 1C). Moreover, we analyzed mRNA microarray data from GSE13507 database, and surprisingly found the mRNA expression of CLCA4 was drastically decreased in the bladder cancer tissues (P<0.001, Figure 1D).

### Correlation of CLCA4 expression with clinicopathological characteristics and prognosis of bladder cancer patients

In order to explore the clinical significance of CLCA4 in bladder cancer, we analyzed the CLCA4 expression pattern in 196 bladder cancer specimens by real-time PCR. The correlation analysis of CLCA4 expression revealed a significant association between CLCA4 expression and tumor size (P = 0.001) and tumor stage (T classification) (P = 0.034) (Table 1). However, there was no significant correlation between CLCA4 expression and other clinicopathologic factors, such as age, sex, or tumor grade. Kaplan–Meier survival analysis revealed that patients with high CLCA4 expression had more favorable overall and recurrent-free survival (P<0.001 and P=0.032, respectively) than those with low CLCA4 expression (Figure 1E). Multivariable Cox regression analyses revealed that low CLCA4 expression was a risk factor for the overall and recurrent-free survival of bladder cancer patients (P=0.001 and P=0.019, respectively) (Table 2).

**Table 1:** Correlation between CLCA4 mRNA expression and clinicopathological characteristics

Characteristic	No.	CLCA4 expression		*P*
		Low	High	
Sex				0.534
Male	121	63	60	
Female	75	35	40	
Age (y)				0.567
≤60	92	48	44	
>60	104	50	54	
Tumor grade				0.884
Low	117	58	59	
High	79	40	39	
Tumor size				**0.001**
<3cm	143	61	82	
≥3cm	53	37	16	
T classification				**0.034**
Ta, T1	130	58	72	
T2-T4	66	40	26	
Total no. of patients	196	98	98	

**Table 2:** Multivariate analysis of overall survival (OS) and Recurrent-free survival (RFS) in patients with bladder cancer

Prognostic variables	OS		RFS	
	Hazard ratio (95% CI)	*P*	Hazard ratio (95% CI)	*P*
Sex (M vs F)				
Age (>60 vs ≤60)				
Tumor grade (High vs Low)				
Tumor size (≥3cm vs<3cm)				
T classification (T2-4 vs T1, Ta)	2.235 (1.254-3.982)	**0.006**		
CLCA4 (High vs Low)	0.338 (0.175-0.654)	**0.001**	0.622 (0.419-0.925)	**0.019**

### CLCA4 suppresses cell viability and growth in bladder cancer

**Figure 2:**
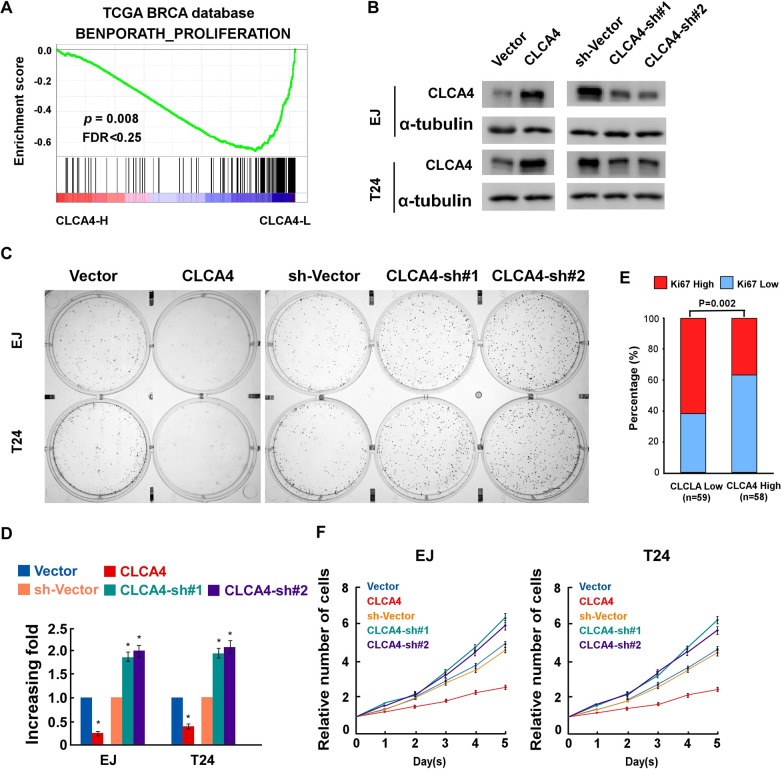
CLCA4 inhibits cell proliferation in bladder cancer cells **(A)** GSEA plots correlating CLCA4 levels with cancer proliferation based on publicly available bladder cancer patient gene expression profiles. **(B)** Western blotting analysis of CLCA4 expression in EJ and T24 cells stably expressing or silencing CLCA4; α-tubulin was used as a loading control. **(C-D)** Representative micrographs (C) and quantification (D) of colonies formed by the indicated cells as determined by colony formation assays. **(E)** The Ki67 mRNA expression levels were negatively related to the CLCA4 expression levels in 196 primary bladder cancer specimens (P=0.002). **(F)** CLCA4 inhibits proliferation rate of bladder cancer cells, as determined by CCK-8 assay. Bar graphs show the statistical analysis of three independent experiments (^*^ P<0.05).

**Figure 3:**
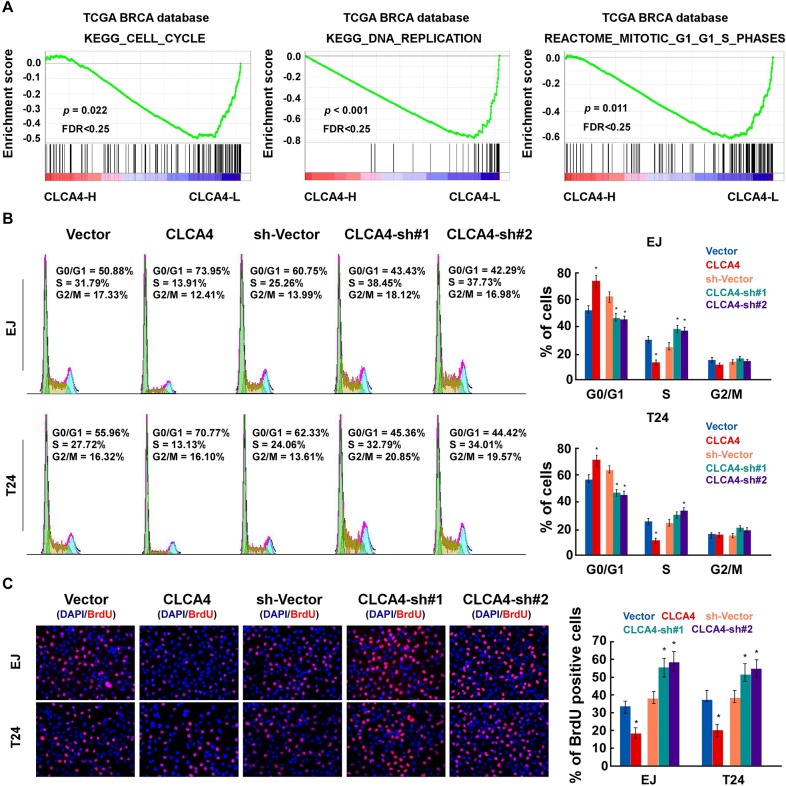
CLCA4 inhibits cell cycle in bladder cancer cells **(A)** GSEA results indicated that CLCA4 expression was significantly correlated with the cell cycle-associated gene signatures. **(B)** Flow cytometric analysis showing the percentages of CLCA4-overexpressing and CLCA4-shRNA transfected cells at different phases of the cell cycle. **(C)** Representative micrographs (left panel) and quantification (right panel) of EdU incorporation in the indicated bladder cancer cells. DAPI was used as a DNA/nuclear stain.

In order to determine whether CLCA4 inhibited the growth capacity of bladder cancer cells, we further explored the biological significance of CLCA4 using Gene Set Enrichment Analysis (GSEA) based on mRNA expression data from the TCGA. The results indicated that low levels of CLCA4 was closely correlated with proliferation-associated gene signature (Figure 2A). We then evaluated the role of CLCA4 on the bladder cancer cell proliferation in established stable CLCA4-overexpressing and CLCA4-knocking down EJ and T24 cells (Figure 2B). CLCA4 overexpression suppressed the colony forming ability of both cell lines (Figure 2C-2D). Additionally, CLCA4 expression was negatively correlated with Ki67 expression from the real-time PCR data (P = 0.002) (Figure 2E). The CCK-8 cell viability assays showed that overexpression of CLCA4 significantly reduced cell viability in both cell lines, while knockdown of CLCA4 had the opposite effect (Figure 2F). Moreover, GSEA results indicated that low CLCA4 expression was associated with cell cycle-associated gene signatures, suggesting that CLCA4 is involved in the cell cycle regulation (Figure 3A). Flow cytometry assay revealed that overexpressing CLCA4 significantly reduced, but silencing CLCA4 increased the percentage of S phase cells (Figure 3B). EdU incorporation assay suggested that the percentages of cells with incorporated EdU was dramatically repressed in CLCA4-overexpressing cells and elevated in CLCA4-silencing cells (Figure 3C). These results implied that CLCA4 may inhibit the cell proliferation in bladder cancer.

### CLCA4 inhibits cell migration and invasion in bladder cancer

**Figure 4:**
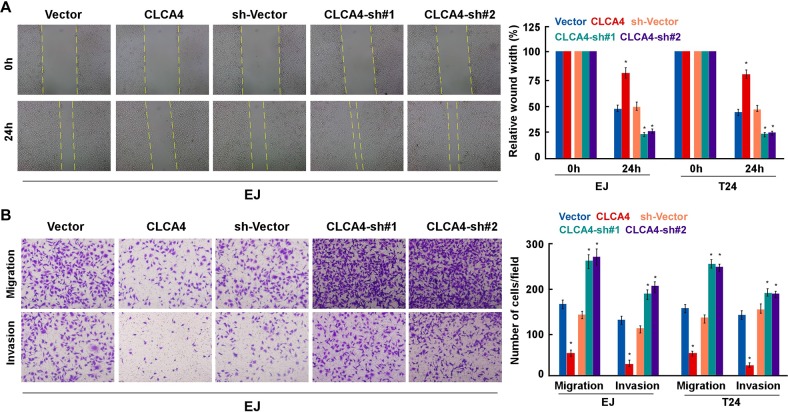
CLCA4 suppresses cell migration and invasion in bladder cancer cells **(A)** The mobility of cells was measured by wound healing assay (left panels), the uncovered areas in the wound healing assays were quantified as a percentage of the original wound area (right panels). **(B)** Effect of CLCA4 overexpression and knockdown in cell migration and invasion was determined by transwell and Matrigel assay (left panels). Quantifications of migrated cells through the membrane and invaded cells through Matrigel of each cell line are shown as proportions of their vector controls (right panels). Bar graphs show the statistical analysis of three independent experiments (^*^ P<0.05).

We next examined the role of CLCA4 in the migration and invasion of bladder cancer cells. The results showed that CLCA4-overexpressing cells exhibited a significantly decreased migratory capacity, whereas CLCA4-knockdown cells had an increased migratory capacity in wound healing assays. Transwell assays showed that the cells on the underside of the filters were remarkably decreased in CLCA4-overexpressing, while increased in CLCA4-knockdown cells (Figure 4B). These results suggest that low expression of CLCA4 attenuate the invasion and migration of bladder cancer cells.

### CLCA4 abrogates tumorigenicity of bladder cancer cells

**Figure 5:**
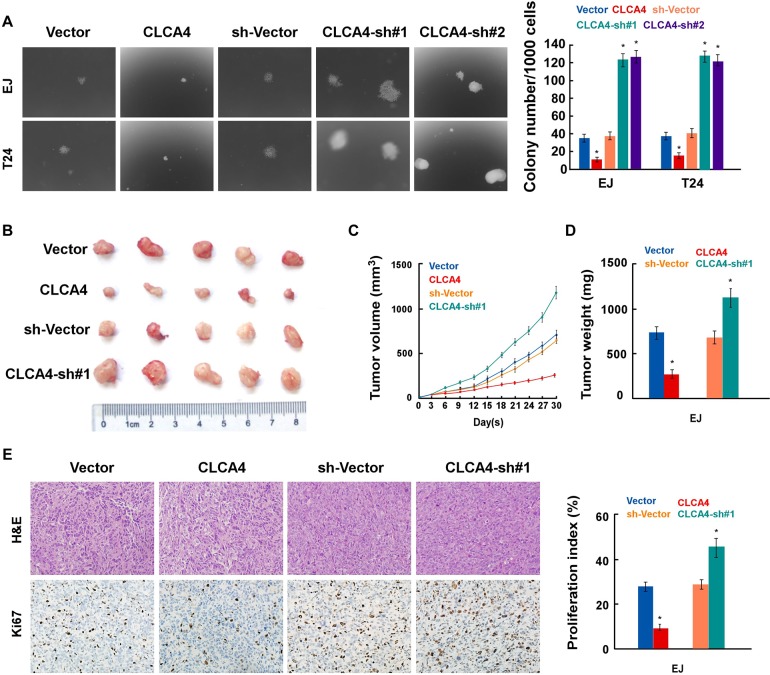
CLCA4 overexpression enhances the tumorigenicity of bladder cancer cells **(A)** Representative micrographs (left) and quantification (right) of colonies formed by the indicated cells, determined by anchorage-independent growth ability assays. **(B)** Representative images of the tumors from Xenograft model in nude mice. **(C)** Tumor volumes were measured on the indicated days. **(D)** Tumor weights of allmice in each group. **(E)** H&E and immunohistochemical staining showed that overexpression of CLCA4 inhibited, whereas suppression of CLCA4 induced the tumorigenicity. Data are mean ± SD of three independent experiments. ^*^P < 0.05.

To investigate the function of CLCA4 on the tumorigenicity of bladder cancer cells, we performed anchorage-independent growth assay. As shown in Figure 5A, CLCA4 overexpressed cells formed a smaller number and smaller colonies compared with control cells, while silence of CLCA4 had the opposite effect. Furthermore, we examined the role of CLCA4 in the tumorigenicity of bladder cancer cells *in vivo*. Consistently, CLCA4-overexpressed tumors were significantly smaller than and weighed less than control cells (Figure 5B–5D). Immunohistochemistry staining assay showed lower expression of Ki67 in CLCA4 overexpressed groups than in the control groups (Figure 5E). Taken together, *in vivo* studies confirmed that depressed expression of CLCA4 promotes tumorigenicity in bladder cancer cells.

### CLCA4 regulates bladder cancer cell proliferation and metastasis via PI3K/AKT signaling pathway and epithelial-mesenchymal transition (EMT)

**Figure 6:**
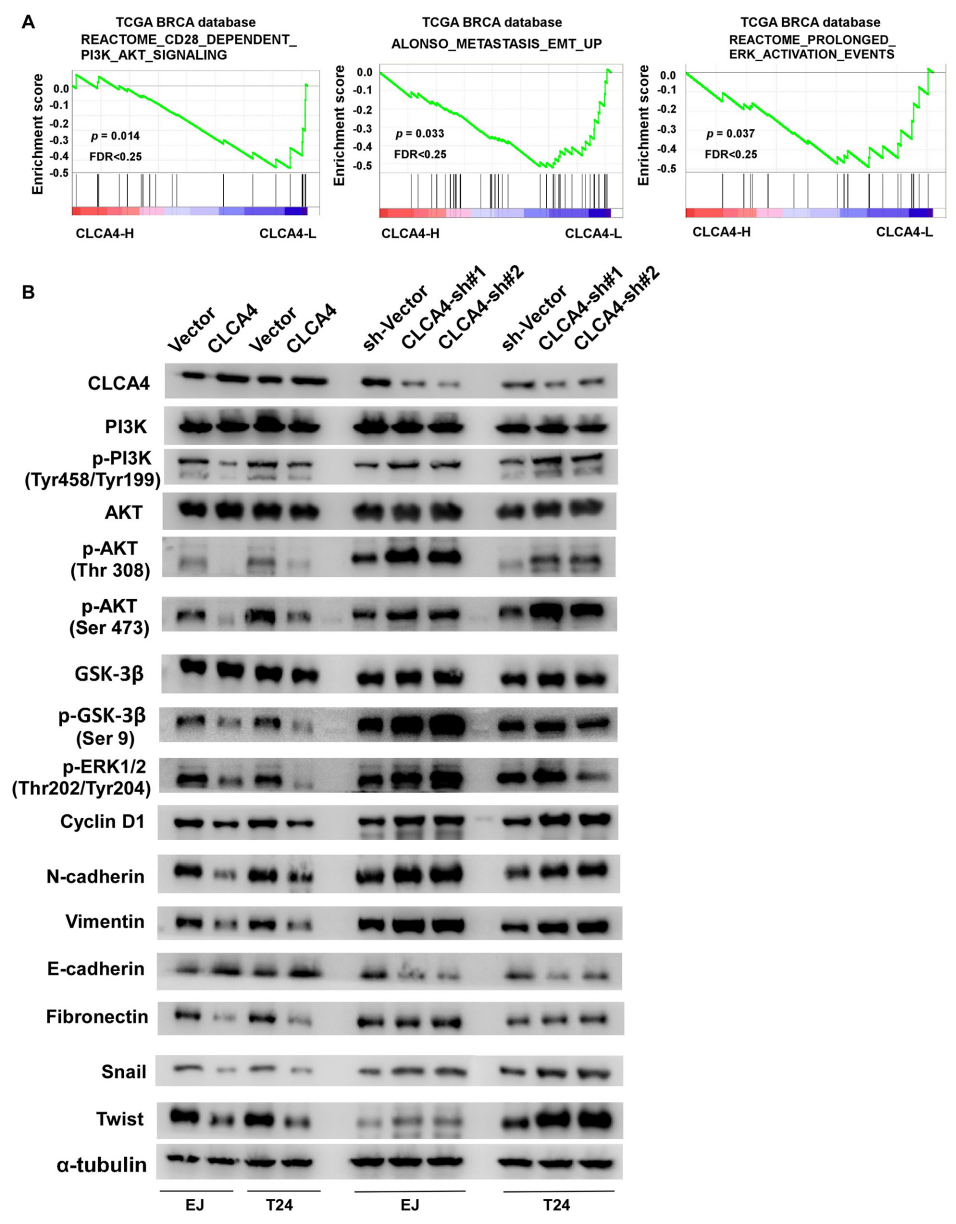
CLCA4 regulates bladder cancer cell proliferation and invasion via PI3K/AKT signaling pathway **(A)** GSEA plot showing that CLCA4 expression negatively correlated with PI3K/AKT, ERK-activated and EMT related gene signatures. **(B)** Protein levels of PI3K, p-PI3K, AKT, p-AKT, GSK3β, p-GSK3β, p-ERK1/2, CyclinD1, epithelial and mesenchymal makers, and transcription factors were shown in indicated cells. α-tubulin was used as a loading control.

**Figure 7:**
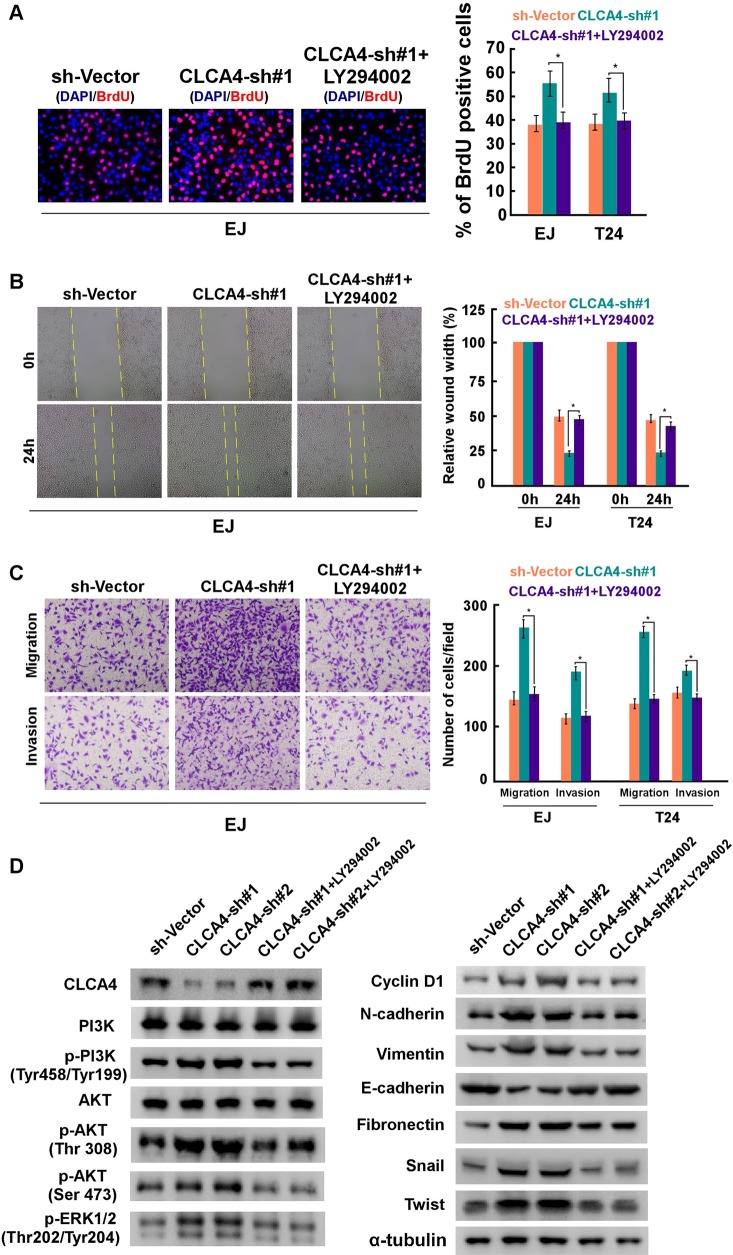
CLCA4 knockdown promoted bladder cancer cell proliferation and invasion are blocked by LY294002 CLCA4-shRNA transfected cells were treated with PI3K inhibitor LY294002, and applied to proliferation, migration and invasion assays. **(A)** Representative images (left panel) and quantification (right panel) of EdU labeling in the indicated cells. **(B)** Representative images (left panel) and quantification (right panel) of migrating cells based on a wound healing assay. **(C)** Representative images (left panel) and quantification (right panel) of invading cells based on transwell and Matrigel assays. Bars represent the mean± SD of three independent experiments. ^*^P< 0.05. **(D)** Protein levels of PI3K, p-PI3K, AKT, p-AKT, p-ERK1/2, epithelial and mesenchymal makers, and transcription factors were compared in indicated cells. α-tubulin was used as a loading control.

To further explore the molecular mechanisms of proliferation, migration and metastasis inhibited by CLCA4 in bladder cancer, GSEA of publicly available gene expression array data was analysed, and we found that CLCA4 expression was negatively associated with the activation of the PI3K/AKT and ERK signaling, and the expression of EMT related genes (Figure 6A). Overexpression of CLCA4 caused a significant decrease in AKT (Thr308, Ser473), GSK3β (Ser9), and ERK1/2(Thr202/Tyr204) phosphorylation. In contrast, phosphorylation of AKT and GSK3β were enhanced in CLCA4-knockdown cells. Additionally, cell-cycle regulator CyclinD1 was obviously down-regulated in CLCA4-overexpressing cells and up-regulation of CyclinD1 was found in CLCA4-knockdown cells as compared with their respective controls (Figure 6B). The results implied that CLCA4 suppresses bladder cancer cell proliferation through the PI3K/AKT signaling pathway. As EMT is one main process of tumor cell migration and invasion, western blot assay was performed to evaluate the effect of CLCA4 on the expression of EMT-related factors. Down-regulation of N-cadherin, vimentin, fibronectin, Zeb1, Twist, and Snail and up-regulation of E-cadherin were observed in CLCA4 overexpressed bladder cancer cells. Conversely, increased N-cadherin, vimentin, fibronectin, Zeb1, Twist, and Snail expression and down-regulation of E-cadherin were detected in CLCA4 silenced cancer cells (Figure 6B). Consistent with these results, we found that PI3K inhibitor LY294002 rescued the suppression of CLCA4 in bladder cancer cell proliferation, migration and invasion (Figure 7A-7D). These results suggested that inactivation of PI3K/AKT signaling pathway by CLCA4 in bladder cancer cells may be the underlying mechanism for its tumor-suppressing effect.

## DISCUSSION

In this study, we found that CLCA4 might play a crucial role in the tumorigenesis and progression of bladder cancer. Clinical analysis unraveled that CLCA4 downregulation in bladder cancer was correlated with poor prognosis of patients with bladder cancer. Downregulation of CLCA4 augmented the growth and invasive abilities of bladder cancer cells, while overexpression of CLCA4 reduced these effects via regulating the PI3K/AKT signaling. Our results provide new insights into the mechanism of CLCA4 deregulation in enhancing development of bladder cancer.

Ion channels are pore-forming transmembrane proteins that regulate the flow of specificions between the extracellular and intracellular environments. As supported by a wide range of evidence, ion channels were frequently involved in many cellular responses key to tumor development, including apoptosis, cell growth, migration, invasion and cell adhesion [16, 17]. An assorted collection of ion channels has also been implicated in bladder cancer progression, while, in most cases, the reported ion channels involved in bladder cancer development are transient receptor potential (TRP) channels. For example, Yamada et al. revealed that TRPV2 channel activation could induce apoptotic cell death of bladder cancer cells [18]. Mizuno et al. showed that TRPM7 and TRPV2 were functionally expressed in bladder cancer cells and functioned as negative regulators of bladder cancer cell proliferation [19]. Remarkably, our study showed that CLCA4 expression was remarkably depressed, and that down-regulation of CLCA4 significantly promoted, while overexpression of CLCA4 profoundly suppressed bladder cancer cell proliferation, colony formation, invasion, and migration. *In vivo* experiments confirmed the tumor suppressor role of CLCA4. Our study firstly revealed the involvement of calcium activated ion channel in bladder cancer progression. These results were supported by previous study in Ca^2+^-activated K^+^ channel [20], in which the authors observed large conductance Ca^2+^-activated K^+^ channels were functionally expressed in bladder cancer cells.

It has been well established that CLCA proteins not only orchestrate inflammatory responses, but also plays an important role in carcinogenesis [4]. Abdel-Ghany et al. demonstrated that CLCA1 can mediate focal adhesion kinase activation and metastatic growth of cancer cells [21]. Additionally, low expression of CLCA1 predicts tumor recurrence and poor survival in colorectal cancer patients [9]. Moreover, CLCA2 was remarkably induced by replicative senescence as well as oxidative stress in a p53-dependent manner in multiple cancer tissues [22, 23]. Collectively, these findings establish a strong relationship between CLCA regulators and tumor development. Nevertheless, the mechanisms by which CLCAs modulates cancer cell proliferation and metastasis during tumor progression are not well established. In this study, we report that inhibition of CLCA4 activated PI3K/AKT signaling pathway and elevated the expression of various AKT downstream genes that specifically regulate cell proliferation, migration, apoptosis, and angiogenesis in bladder cancer. PI3K/AKT signaling has been shown to play a pivotal role in the proliferation and invasion of cancer cells [24], and the importance of PI3K/AKT signaling in bladder cancer progression has been implicated [25]. Activation of the PI3K/Akt signaling has also been showed to be correlate with tumour progression and clinical survival in bladder cancer patients [26]. However, the biological relationship of CLCA4 and PI3K/Akt signaling in bladder cancer progression remains unclear. To our knowledge, this is the first mechanistic study providing definitive evidence for the involvement of CLCA protein in human cancer development.

Notably, CLCA proteins have been reported to be closely associated with the prognosis of cancerpatients. Yang et al. demonstrated that CLCA1 expression levels were significantly correlated with the tumor grade, lymph node metastasis, and overall survival rate in patients with colorectal cancer [9]. Man et al. reported that the increased detection of CLCA2 in circulating tumor cells predict shorter survival time in lung adenocarcinoma patients [27]. Recently, Yu et al. found that low expression of CLCA4 in basal and luminal B breast cancer patients was correlated with lower relapse-free survival [13]. Unlike their results, our study showed that CLCA4 could be a potential predictor for both overall and recurrent-free survival in bladder cancer patients. Moreover, we showed that CLCA4 expression were significantly correlated with tumor stage and tumor size. Collectively, these studies suggest that CLCA proteins are useful indicators for clinical prognosis in human cancers.

In conclusion, the present study indicated a inverse correlation between CLCA4 expression and the clinical prognosis of bladder cancer patients. Downregulation of CLCA4 augmented bladder cancer growth and metastasis *in vitro* and *in vivo*, which was assumed to be acted by activation PI3K/AKT signaling. Therefore, CLCA4 might be exploited as a target for potential anti-cancer treatment of bladder cancer.

## MATERIALS AND METHODS

### Cell culture

Bladder cancer cell lines (T24, 5637, BIU-87, UM-UC-3, RT4, EJ, TCCSUP) and uroepithelial cell SV-HUC-1 was purchased from the Cell Bank of the Chinese Academy of Sciences. Primary normal bladder urothelial cells (NBUCs) cultures and human uroepithelial cell SV-HUC-1 were established as described previously [14]. The cell lines EJ, 5637, BIU-87, UM-UC-3, T24, and TCCSUP were maintained in RPMI 1640 medium, while the RT4 cell line was cultured in McCoy's 5a medium supplemented with 10% fetal bovine serum (FBS) (Gibco). SV-HUC-1 cells was grown in F12K medium supplemented with 10% FBS.

### Cell counting kit-8 (CCK-8) assay

Cell viability was quantified by using the CCK-8 kit (DOJINDO Laboratories, Japan) as previously described [15]. Cells were plated on 60 mm plates (0.5×10^3^ cells/plate) and cultured for 10 days. Colonies were then fixed and stained with 1mg/ml crystal violet.

### Wound-healing assay

Cells were grown to complete confluence, and then a wound was made with a sharp edge to make a cell-free area on each confluent monolayer. The wound was observed and photographed, and the gap of the scraped area was measured at 0 and 24 h.

### Migration and invasion assays

The capacities for cell migration and invasion were evaluated by using transwell chambers (Corning LifeSciences). After pretreated, 1×10^5^ cells suspended in 100 ml of serum-free medium were seeded in the upper chamber of the Transwell system, and medium supplemented with 10% FBS was added to the lower chamber. For invasion assay, Cell were seeded in pre-coated Matrigel transwell insert chambers. After incubation for 24 h, cells remaining on the top surface were removed and cells migrated to the lower surface of the membrane were fixed and stained with 0.1% crystal violet.

### Tissue samples

A total of 196 bladder cancer patients diagnosed at The Second Affiliated Hospital of Nanchang University were selected for this study. The stages of all samples were determined according to the American Joint Committee on Cancer (AJCC)'s classification system on TNM staging. The tumor grade was determined according to the World Health Organization/ International Society of Urological Pathologists criteria. Prior patients' consents were obtained and approval was obtained from the ethics committee of The Second Affiliated Hospital of Nanchang University. The five years follow-up period concluded in December 2012, and 47 patients died and 101 patients have recurrent diseases. The median survival time is 52 months. In addition, 60 pairs of snap-frozen bladder cancer and adjacent non-tumor samples were obtained. To select adjacent non-tumor bladder tissues, grossly normal mucosa from the farthest resection margin was carefully excised and subjected to frozen section evaluation in order to exclude dysplasia and the presence of carcinoma cells. The urothelium and submucosal layers of an adjacent area was then carefully peeled off and placed immediately in liquid nitrogen.

### Quantitative real-time PCR (qRT-PCR)

Total RNA from tissue samples and cells was extracted using the TRIzol reagent kit (Invitrogen, California, USA). Reverse transcription–PCR was performed using the RevertAid First Strand cDNA Synthesis kit (Thermo, Massachusetts, USA). Real-time quantitative PCR was performed on a StepOne Plus real-time PCR system (Life Technologies, Carlsbad, CA, USA). The sequences of primers were provided in the Supplementary Table 1. GAPDH was used as an internal control.

### Western blotting

Cells and tissues were lysed with RIPA Lysis Buffer, and cleared by centrifugation at 4°C. Protein concentration was determined by Bradford assay (Thermo Scientific, Massachusetts, USA). Equal amounts of protein were separated by SDS-PAGE gels, and then transferred to polyvinylidene difluoride membrane, and probed with corresponding antibodies: CLCA4 (cat. no. ab197347), CyclinD1 (cat. no. ab134175), E-cadherin (cat. no. ab76319), N-cadherin (cat. no. ab76057), Vimentin (cat. no. ab137321), Fibronectin (cat. no. ab2413) (1:1000; Abcam, USA), p-PI3K(Tyr458/Tyr199) (cat. no. 4228), PI3K (cat. no. 4255), p-AKT(Thr 308) (cat. no. 4056), p-AKT(Ser 473) (cat. no. 4051), AKT (cat. no. 9272), p-GSK-3β(Ser 9) (cat. no. 9336), GSK-3β (cat. no. 9832), p-ERK1/2(Thr202/Tyr204) (cat. no. 9101), Zeb1 (cat. no. 3396), Snail (cat. no. 3895), Twist (cat. no. 46702) (1:1000; Cell signaling, Boston, MA, USA), and α-tubulin (cat. no. sc-5286) (1:2000; Santa Cruz, USA).

### Immunohistochemistry

Formalin-fixed, paraffin-embedded mice tumor specimens were cut into 4-μm thick sections for immunohistochemical staining. Sections were deparaffinized in xylene and rehydrated, and were then treated with 3% hydrogen peroxide in deionized water. Tissue sections were immunostained with monoclonal mouse anti-Ki67 antibody (cat. no. ab16667) (Abcam, Cambridge, USA; 1:150) at 4°C overnight, and incubated with a biotinylated secondary antibody (Abcam, Cambridge, USA), followed by further incubation with 3,3-diaminobenzidine tetrahydrochloride (DAB) and counterstain with haematoxylin. Proliferation index was quantized by counting proportion of Ki67-positive cells among the total number of invasive cells in the area scored.

### Plasmids, lentiviral infection, and transfection

Human CLCA4 cDNA was amplified by PCR and cloned into GV358 lentiviral vector (GeneChem, Shanghai, China). Oligo of CLCA4 shRNAs was synthesized and inserted in GV248 vector Genechem Co., Ltd (GeneChem, Shanghai, China). Stable cell lines that expressed CLCL4 (CLCA4) or CLCA4-shRNAs (CLCA-sh#1 and CLCA-sh#2) were selected for 10 days with 0.5 mg/ml puromycin. In some experiments, cells were treated with LY294002 (50 nM) for 12 h before harvest.

### Flow cytometry analysis

Cells were harvested, washed, and fixed with 75% alcohol, followed by incubation with 2 μg/ml Bovine pancreatic RNAase (Sigma-Aldrich) at 37°C for 30 min. Then, the cells were incubated in 20 mg/ml of propidium iodide (Sigma-Aldrich; USA) at room temperature for 20 min. Cell cycle was analyzed by using a BD LSRFortessa X-20 instrument (BD Biosciences, CA, USA).

### EdU Labeling

Cells were incubated with 5-ethynyl-2′-deoxyuridine (EdU, RiboBio; R11053) for 3 h at 37°C, and treated with ApolloR reaction cocktail according to the manufacturer's instructions. Images were collected using a fluorescent microscopy (Olympus, Japan).

### Anchorage-independent growth ability assay

The cells were trypsinized and counted. 5× 10^3^ cells were mixed with complete medium containing 0.3% agar (Sigma-Aldrich; USA) on 6-well plate, followed by plating on top of a bottom layer with 1% agar completed medium mixture. After 10 days incubation, viable colonies larger than 0.1 mm in diameter were scored.

### Tumour xenografts

All experimental procedures were approved by the Institutional Animal Care and Use Committee of Tongji Medical College of Huazhong University of Science and Technology. BALB/c nude mice (5–6 weeks old) were purchased from the Center of Experimental Animal of Tongji Medical College of Huazhong University of Science and Technology and randomized into groups. For tumor formation assay, 5×10^6^ cells was injected subcutaneously into a single side of each mouse. Tumor volume was calculated using the following equation: length × (width)2/2. Mice were sacrificed after 30 days, and the tumors were detected by an IVIS imagining system (Caliper).

### Statistical analysis

All statistical analyses were carried out using SPSS 16.0 software. Significance was analyzed by Student's t-test. The correlation between CLCA4 expression and clinicopathologic characteristics was analyzed using Pearson's chi-square test. The cut-point of CLCA4 and Ki67 genes mRNA expression was defined as the median. Survival curves were plotted by the Kaplan–Meier method and compared using the log-rank test. P <0.05 was considered statistically significant. Data are expressed as mean ± SD from at least 3 independent experiments.

## SUPPLEMENTARY MATERIALS TABLE



## Abbreviations

CLCA4: Calcium activated chloride channel A4
TRP: transient receptor potential
CCK-8: Cell counting kit-8
AJCC: American Joint Committee on Cancer
