# Correction: CLCA4 inhibits bladder cancer cell proliferation, migration, and invasion by suppressing the PI3K/AKT pathway

**DOI:** 10.18632/oncotarget.26656

**Published:** 2019-01-29

**Authors:** Teng Hou, Lijie Zhou, Longwang Wang, Gallina Kazobinka, Xiaoping Zhang, Zhaohui Chen

**Affiliations:** ^1^ Department of Urology, Union Hospital, Tongji Medical College, Huazhong University of Science and Technology, Wuhan 430022, China; ^2^ Department of Urology, The Second Affiliated Hospital of Nanchang University, Nanchang, Jiangxi 330008, China

**This article has been corrected:** The correct affiliation information is given below:

^**1**^ Department of Urology, Union Hospital, Tongji Medical College, Huazhong University of Science and Technology, Wuhan 430022, China

Original article: Oncotarget. 2017; 8:93001-93013. https://doi.org/10.18632/oncotarget.21724

