# Supplementary material for: CLCA4 inhibits bladder cancer cell proliferation, migration, and invasion by suppressing the PI3K/AKT pathway
Source: Oncotarget. 2017 Oct 9;8(54):93001–13. doi: 10.18632/oncotarget.21724 (PMC5696239; doi:10.18632/oncotarget.21724)
Supplement: Supplementary file 1 [file oncotarget-08-93001-s001.pdf]

CLCA4 inhibits bladder cancer cell proliferation, migration, and invasion by suppressing the PI3K/AKT pathway

SUPPLEMENTARY MATERIALS

Supplementary Table 1: qRT-PCR primers sequences

Gene	Forward Primer (5′–3′)	Reverse Primer (5′–3′)
CLCA4	TTTGGGGCTCTTACATCAGG	GTGTCGTTCATCCAGGCATT
Ki67	TCCTTTGGTGGGCACCTAAGACCTG	TGATGGTTGAGGTCGTTCCTTGATG
CCND1	GCTGCGAAGTGGAAACCATC	CCTCCTTCTGCACACATTTGAA
N-cadherin	TGCGGTACAGTGTAACTGGG	GAAACCGGGCTATCTGCTCG
E-cadherin	ATTTTTCCCTCGACACCCGAT	TCCCAGGCGTAGACCAAGA
VIM	AGTCCACTGAGTACCGGAGAC	CATTTCACGCATCTGGCGTTC
a-SAM	CGGGACTAAGACGGGAATCC	CCTTACAGAGCCCAGAGCCATTG
ZEB1	CAGCTTGATACCTGTGAATGGG	TATCTGTGGTCGTGTGGGACT
snail	ACTGCAACAAGGAATACCTCAG	GCACTGGTACTTCTTGACATCTG
slug	TGTGACAAGGAATATGTGAGCC	TGAGCCCTCAGATTTGACCTG
GAPDH	TCAAGAAGGTGGTGAAGCAG	CGTCAAAGGTGGAGGAGTG
